# Immunohistochemistry-Based Molecular Classification of Muscle-Invasive Bladder Urothelial Carcinoma: Survival Trends Across Molecular Subtypes

**DOI:** 10.3390/ijms27188082

**Published:** 2026-09-11

**Authors:** Andrada-Claudia Tătar, Septimiu Voidăzan, Andrada Raicea, Maria-Cătălina Popelea, Andrada Loghin, Angela Borda

**Affiliations:** 1 Histology Department, George Emil Palade University of Medicine, Pharmacy, Science, and Technology of Targu Mures, 540142 Targu Mures, Romania; andrada-claudia.tatar@umfst.ro (A.-C.T.); andrada.raicea@umfst.ro (A.R.); maria-catalina.popelea@umfst.ro (M.-C.P.); andrada.loghin@umfst.ro (A.L.); angela.borda@umfst.ro (A.B.); 2 Doctoral School of Medicine and Pharmacy, George Emil Palade University of Medicine, Pharmacy, Science, and Technology of Targu Mures, 540142 Targu Mures, Romania; 3 Pathology Department, Mures Clinical County Hospital, 540011 Targu Mures, Romania; 4 Epidemiology Department, George Emil Palade University of Medicine, Pharmacy, Science, and Technology of Targu Mures, 540142 Targu Mures, Romania; 5 Pathology Department, Targu Mures Emergency Clinical County Hospital, 540136 Targu Mures, Romania

**Keywords:** muscle-invasive bladder urothelial carcinoma, molecular classification, immunohistochemistry

## Abstract

Muscle-invasive bladder urothelial carcinoma (MIBUC) is a heterogeneous disease, presenting variable clinical outcomes and therapeutic responses. Although molecular classification provides important prognostic information, its clinical use is limited by cost and technical complexity, making immunohistochemistry (IHC) a practical and reliable surrogate approach. We aimed to investigate the association between IHC-based molecular subtypes and overall survival (OS). We conducted a retrospective study including 75 newly diagnosed MIBUCs over a 4-year period. Tumours were stratified into seven molecular subtypes (LumP, LumNS, LumU, Ba/Sq, NE-like, stroma-rich, and double-positive) using a five-antibody IHC panel (GATA3, CK5/6, p16, FGFR3, and EpCAM). Marker expression was evaluated based on staining intensity and the proportion of positive tumour cells, using predefined, marker-specific cut-off values. OS was assessed for each subtype and compared across groups. Median OS ranged from 5 months (NE-like) to 26 months (stroma-rich and double-positive). LumU demonstrated the highest 1-year survival rate, while the double-positive subtype exhibited the most favourable long-term outcomes, at 2 and 5 years. Conversely, NE-like had the lowest 1-year survival rate, whereas the stroma-rich subtype demonstrated the lowest 2- and 5-year survival rates. No statistically significant difference in OS was identified across molecular subtypes (*p* = 0.714). Patients ≤65 years experienced significantly longer OS than older patients (*p* = 0.024). OS was comparable between sexes (*p* = 0.626) and did not differ significantly according to tumour morphology (*p* = 0.884). IHC-based molecular classification represents a feasible approach to characterize molecular heterogeneity in MIBUC, with further studies needed to clarify its prognostic significance.

## 1. Introduction

Muscle-invasive bladder urothelial carcinoma (MIBUC) represents approximately 20% of all newly diagnosed bladder tumours [1,2] and is characterised by an aggressive clinical profile, with a 5-year survival rate of about 50–60% [3]. MIBUC encompasses diverse histological subtypes (e.g., micropapillary, nested, plasmacytoid, and sarcomatoid) and may exhibit divergent squamous or glandular differentiation. Importantly, urothelial carcinoma with squamous or glandular differentiation should be distinguished from primary squamous cell carcinoma and adenocarcinoma of the bladder, respectively, as the former retain a conventional urothelial component, whereas the latter represent primary non-urothelial malignancies [2]. Accurate diagnosis of MIBUC is established by histopathological examination of transurethral resection of the bladder tumour (TURBT) specimens, which remains the current gold standard diagnostic approach [4]. The standard treatment protocol involves radical cystectomy (RC) with pelvic lymph node dissection, often integrated with cisplatin-based neoadjuvant chemotherapy (NAC), which provides a 5% improvement in cancer-specific survival (CSS) at 5 years [5,6]. Despite this aggressive management, patient outcomes remain highly variable, largely owing to the marked heterogeneity of MIBUC, which reflects substantial differences in tumour biology [7,8].

These biological differences are manifested in distinct patterns of tumour behaviour and therapeutic response, highlighting the intrinsic diversity among tumours. To better characterise this heterogeneity and its clinical implications, several molecular classifications have been proposed based on tumour transcriptome analysis using mRNA expression profiling, identifying between two and seven molecular subtypes [9,10,11,12,13,14]. Building on these studies, a consensus molecular classification was subsequently established, defining six molecular subtypes: three luminal (luminal papillary—LumP, luminal non-specified—LumNS, luminal unstable—LumU), one basal/squamous—Ba/Sq, one neuroendocrine-like—NE-like, and one stroma-rich [15]. The clinical relevance of these molecular subtypes is reflected in their distinct prognostic and predictive characteristics. Specifically, the basal subtype is associated with worse overall survival (OS) compared with the luminal group, yet it shows improved outcomes with NAC, while enhanced responses to immune checkpoint inhibitors have been reported in certain luminal subtypes [16,17,18]. These findings underscore the dual role of molecular classification in predicting patient outcomes and guiding treatment strategies.

Currently, transcriptome-based molecular classification is not routinely implemented in clinical practice due to its relatively high cost and technical complexity. As a practical alternative, immunohistochemistry (IHC) is widely available, reliable, and cost-effective, and can be used to classify tumours into molecular subtypes. Recent studies have evaluated a wide range of IHC markers for this purpose, aiming to develop a validated, standardized antibody panel. While a fully validated panel is not yet available, evidence from the most robust published studies supports the use of a five-antibody panel (GATA3, CK5/6, p16, FGFR3—fibroblast growth factor receptor 3, and EpCAM—epithelial cell adhesion molecule) to classify tumours at the time of diagnosis. Therefore, the initial classification is based on the dual GATA3 and CK5/6 IHC profile, which categorizes tumours as luminal (GATA3+, CK5/6−), Ba/Sq (GATA3−, CK5/6+), double-positive, or double-negative. Within the luminal group, p16 expression distinguishes the LumP (p16−) subtype from the LumNS and LumU (p16+) subtypes, while FGFR3 expression further differentiates LumNS (FGFR3+) from LumU (FGFR3−). Among double-negative tumours, EpCAM+ defines the NE-like subtype, while EpCAM− tumours are classified as stroma-rich [19].

In light of growing evidence supporting the potential clinical utility of IHC-based molecular classification in MIBUC, further studies are warranted to evaluate its clinical utility in routine practice. Therefore, this study aimed to assess survival differences among consensus molecular subtypes through analysis of OS across subtypes, thereby contributing to improved prognostic stratification and potentially supporting treatment decision-making at diagnosis. To achieve this, tumours were classified into molecular subtypes using a previously proposed IHC marker panel as a surrogate method. To the best of our knowledge, this represents the first application of an IHC-based classifier encompassing all consensus molecular subtypes in the context of survival analysis.

## 2. Results

### 2.1. Clinicodemographic Characteristics of the Cohort

A total of 75 patients with MIBUC were included in the study, with a median age of 71.0 years (IQR, 64.0–78.5). The majority of patients (72.0%) were older than 65 years at diagnosis. The cohort showed a marked male predominance, comprising 69 men and 6 women (male-to-female ratio, 11.5:1).

Metastatic disease was documented at the time of initial diagnosis in 1 patient (1.3%). Regarding subsequent definitive treatment, 8 of the 75 patients (10.7%) underwent radical cystectomy, while 1 patient (1.3%) underwent partial cystectomy.

### 2.2. Pathological Characteristics of the Tumours

All tumours were pT2 and of high histological grade. Conventional urothelial morphology was identified in 55 cases (73.3%), whereas 20 cases (26.7%) exhibited variant histology. Among these, 11 cases (14.7%) showed squamous differentiation, 2 cases (2.7%) showed glandular differentiation, and 4 cases (5.3%) showed micropapillary subtype, while the nested, plasmacytoid, and sarcomatoid subtypes were each identified in one case (1.3%).

### 2.3. Molecular Subtypes

**Figure 1:**
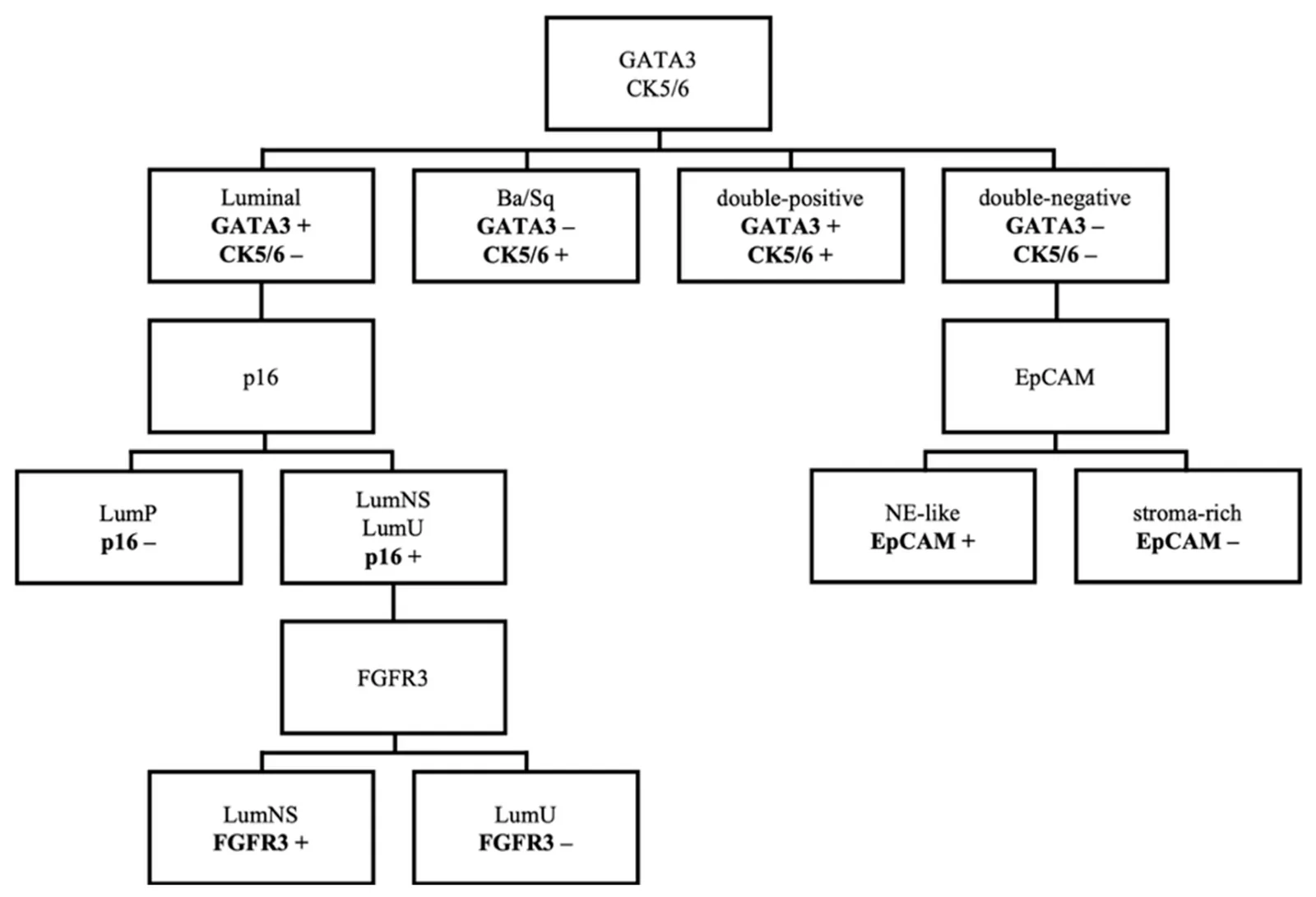
Algorithm for the assignment of MIBUC cases to IHC-defined molecular subtypes.

Based on IHC analysis, tumours were classified into seven molecular subtypes according to the predefined IHC algorithm described above, illustrated in Figure 1. Luminal subtypes collectively represented the largest category, with LumU (21.3%), LumP (20.0%), and LumNS (17.3%) accounting for nearly 60% of all cases. The double-positive subtype was identified in 18.7% of cases, whereas the Ba/Sq, NE-like, and stroma-rich subtypes were less frequent, together comprising approximately one-quarter of the cohort. Median age at diagnosis varied modestly across molecular subtypes, ranging from 65.5 years in the LumU subtype to 74.0 years in the stroma-rich subtype. Female patients were identified only in the LumP, LumU, Ba/Sq, and double-positive groups, whereas LumNS, NE-like, and stroma-rich tumours occurred exclusively in men. Conventional UC predominated across most molecular subtypes, whereas variant histology was proportionally more frequent in the Ba/Sq and double-positive subtypes. However, these differences did not reach statistical significance (*p* = 0.17). Cohort characteristics according to molecular subtypes are summarised in Table 1.

### 2.4. Overall Survival Analysis

Thirteen patients (17.3%) were alive at the end of the follow-up period, with fewer than one in five patients remaining alive at 5 years. Among the 9 patients who underwent cystectomy, 7 had died by the end of follow-up, whereas 2 remained alive. The overall median survival of the study cohort was 16 months (95% CI, 7.5–24.5).

**Figure 2:**
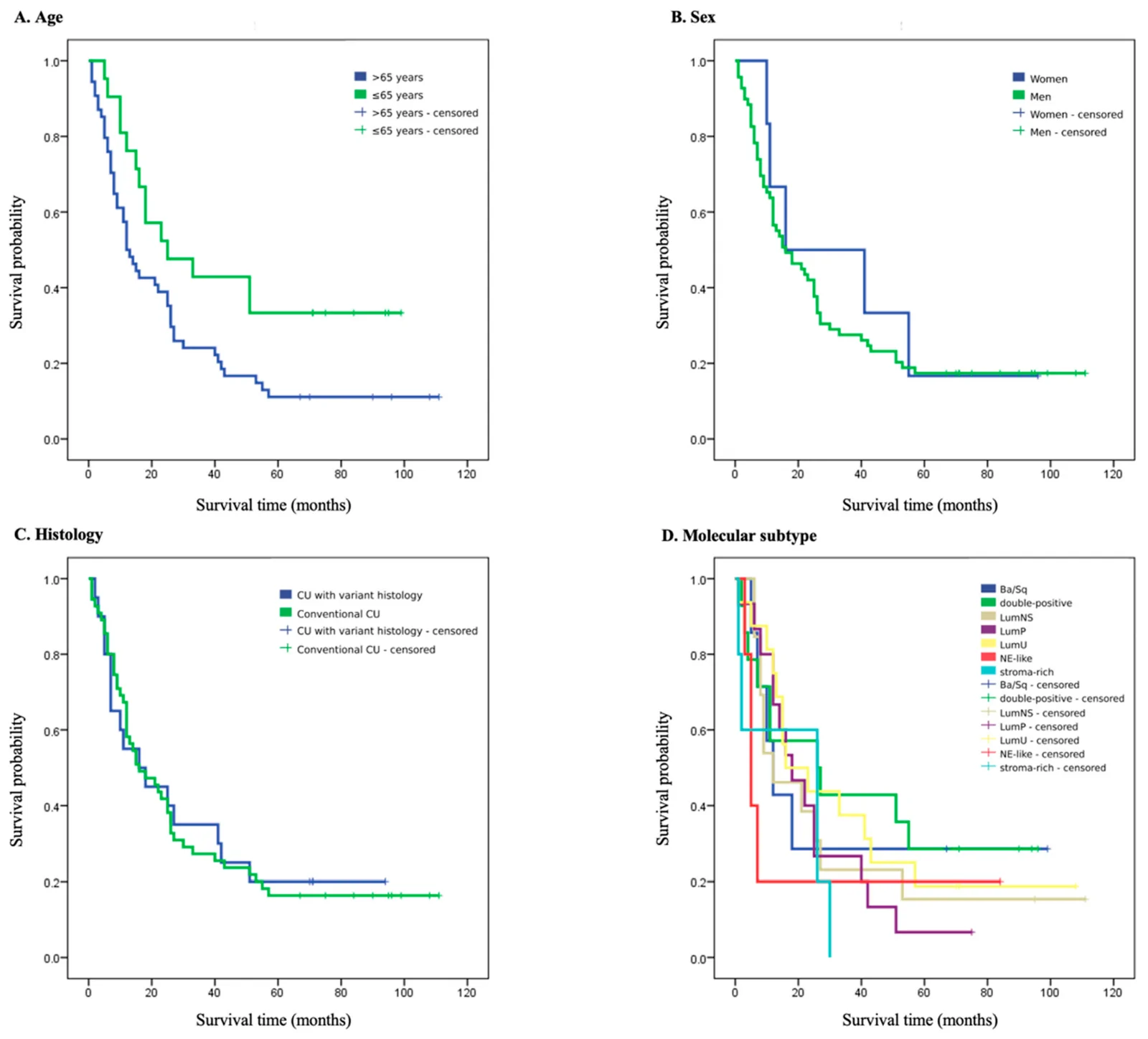
(**A**) Kaplan–Meier analysis of overall survival (OS) by age (≤65 vs. >65 years); (**B**) Kaplan–Meier analysis of OS by sex; (**C**) Kaplan–Meier analysis of OS by histology; (**D**) Kaplan–Meier analysis of OS by molecular subtypes.

Patients ≤65 years had a significantly longer OS than those >65 years, with a median survival of 25 months versus 12 months, respectively (*p* = 0.024). This difference was reflected by consistently higher estimated survival rates at 1, 2, and 5 years in younger patients (76.2% vs. 50.0%, 47.6% vs. 35.2%, and 33.3% vs. 11.1%, respectively). OS was comparable between men and women, with a median OS of 16 months in both groups (*p* = 0.626). Although estimated survival rates were similar throughout follow-up, women showed slightly higher survival rates at both 1 and 2 years after diagnosis. OS did not differ significantly between patients with conventional UC and those with variant histology (*p* = 0.884). The corresponding 1-, 2-, and 5-year survival rates according to age, sex, and histology are summarised in Table 2 and illustrated in Figure 2A–C.

Median OS varied across the molecular subtypes, ranging from 5 months in the NE-like subtype to 26 months in both the stroma-rich and double-positive subtypes. The LumU subtype exhibited the highest estimated 1-year survival rate, whereas the double-positive subtype showed the most favourable long-term outcome, with the highest estimated 2- and 5-year survival rates. Conversely, the NE-like subtype had the lowest estimated 1-year survival rate, while the stroma-rich subtype demonstrated the lowest estimated 2- and 5-year survival rates. Detailed survival estimates across molecular subtypes are presented in Table 3, and the corresponding Kaplan–Meier curves are shown in Figure 2D. No statistically significant differences in OS were observed among the seven molecular subtypes (log-rank *p* = 0.714).

## 3. Discussion

To the best of our knowledge, this is the first study to comprehensively apply an IHC-based classifier encompassing all consensus molecular subtypes of MIBUC in the context of survival analysis. Molecular classification of MIBUC remains an active area of research, driven by the need to improve our understanding of tumour biology and to facilitate more individualized therapeutic strategies [18,20,21]. Although IHC-based approaches have emerged as accessible and cost-effective surrogate tools for molecular subtyping [18,22,23,24,25,26,27,28,29,30,31,32], a fully standardized and universally validated antibody panel has yet to be established. Within this context, the present study applied a previously proposed IHC-based surrogate panel to classify tumours into the seven molecular subtypes of MIBUC at the time of diagnosis and evaluated their association with OS.

Our findings corroborate the well-established predominance of older age and male sex in MIBUC, in agreement with previously published data [2,33]. Nomikos et al. reported a median age at diagnosis of 70 years, which closely aligns with our cohort (71 years) [34]. Similarly, Albakri et al. demonstrated a marked male predominance (88.6%), comparable to that observed in our study (92%) [35]. Regarding tumour histology, pure UC was the most frequent entity in our cohort (73.3%), consistent with the majority of published reports. In our cohort, 17.3% of patients were alive at the end of the 5-year follow-up period, comparable to the 27% survival rate reported in the literature [36].

Our study demonstrated longer OS in patients younger than 65 years, in agreement with previously published studies reporting improved outcomes in younger patients [37]. In our cohort, a trend toward higher estimated survival was observed in women; however, this difference was not statistically significant and was consistent with previous studies reporting no survival differences according to sex [37]. In contrast, although de la Calle et al. reported improved OS in patients with pure UC, our analysis did not demonstrate a significant difference in survival between conventional UC and UC with variant histology [37].

The highest 1-year survival rates were observed in the LumU (75%) and LumP (66.7%) subtypes. Although direct luminal subclassification is not consistently reported in the literature, several studies have demonstrated improved survival outcomes in the luminal category as a whole [22,23,26,31]. In particular, Labban et al. reported improved disease-specific survival (DSS) in luminal compared with double-positive tumours, which is consistent with our findings at 1 year [38].

The Ba/Sq subtype demonstrated intermediate 1- and 2-year survival rates (42.9% and 28.6%, respectively). In agreement with our results, Ying et al. also reported a poorer prognosis for basal tumours compared with luminal subtypes [25]. Similarly, Gupta et al. described early mortality in basal tumours, although they observed comparable long-term OS between luminal and basal subtypes at 6 years, in contrast to our findings [26].

The lowest 1-year survival was observed in the NE-like subtype, whereas the stroma-rich subtype showed the poorest 2- and 5-year survival outcomes. Although previous studies have not separately analysed NE-like and stroma-rich tumours within the double-negative category, several reports have consistently associated this group with the worst OS among molecular subtypes [18,30,31,32].

The double-positive subtype demonstrated the most favourable survival at 2 and 5 years, in line with findings reported by Rashad et al., who also observed the highest OS in this subgroup [22]. Similarly, Bejrananda et al. reported improved 5-year survival in tumours co-expressing GATA3 and CK5/6 [30]. Overall, survival differences across molecular subtypes did not reach statistical significance, consistent with several previously published studies [20,24,27,28,29].

Further studies in larger patient cohorts are warranted to validate our findings. In addition, a standardized and validated IHC antibody panel is required to ensure reproducibility and facilitate broader implementation of this approach.

From a clinical perspective, the proposed IHC-based algorithm represents a practical and cost-effective strategy for molecular stratification in routine practice. Because it relies on widely available biomarkers, it may facilitate the implementation of molecular classification in centres where transcriptomic profiling is not readily accessible, thereby improving tumour characterization and supporting future patient stratification.

Our study has several limitations. First, although OS is a robust and widely used endpoint, we were unable to assess recurrence-free survival, progression-free survival, or CSS due to incomplete follow-up data, including loss to follow-up, transfer of care to other institutions, and, importantly, the lack of reliable information on the cause of death. Second, the relatively small sample size limited the statistical power of our analyses. Finally, although molecular subtyping of bladder cancer is an active area of research, it is not yet implemented in routine clinical decision-making, as current evidence remains inconsistent and is derived predominantly from retrospective studies.

## 4. Material and Methods

### 4.1. Study Design and Case Selection

We conducted a retrospective study over a 4-year period (January 2017–December 2020), including newly diagnosed MIBUC cases from the Pathology Departments of the Târgu Mureș Emergency Clinical County Hospital and the Mureș Clinical County Hospital, Târgu Mureș, Romania, all of which were diagnosed on TURBT specimens. Patient-related information was obtained from hospitals’ electronic medical records. Demographic and clinical data, including age, sex, date of diagnosis, and date of death (if applicable), were collected for each case. Missing survival data were obtained through telephone contact with patients or their next of kin.

The study was approved by the Ethics Committee of George Emil Palade University of Medicine, Pharmacy, Science, and Technology of Târgu Mureș (letter of approval no. 3805/06.06.2025).

### 4.2. Pathological Data

All original hematoxylin and eosin (H&E) slides were reviewed by two pathologists with expertise in uropathology (AL, AB) to reconfirm the diagnosis and the tumour stage, in accordance with the latest editions of the WHO Classification of Urinary and Male Genital Tumours [39] and the AJCC Cancer Staging Manual [40]. The most representative formalin-fixed paraffin-embedded (FFPE) block containing adequate muscle-invasive tumour tissue was selected for IHC analysis.

### 4.3. Inclusion and Exclusion Criteria

The study cohort comprised only patients with MIBUC at initial presentation, diagnosed on TURBT specimens, with sufficient tumour tissue available for IHC analysis.

**Figure 3:**
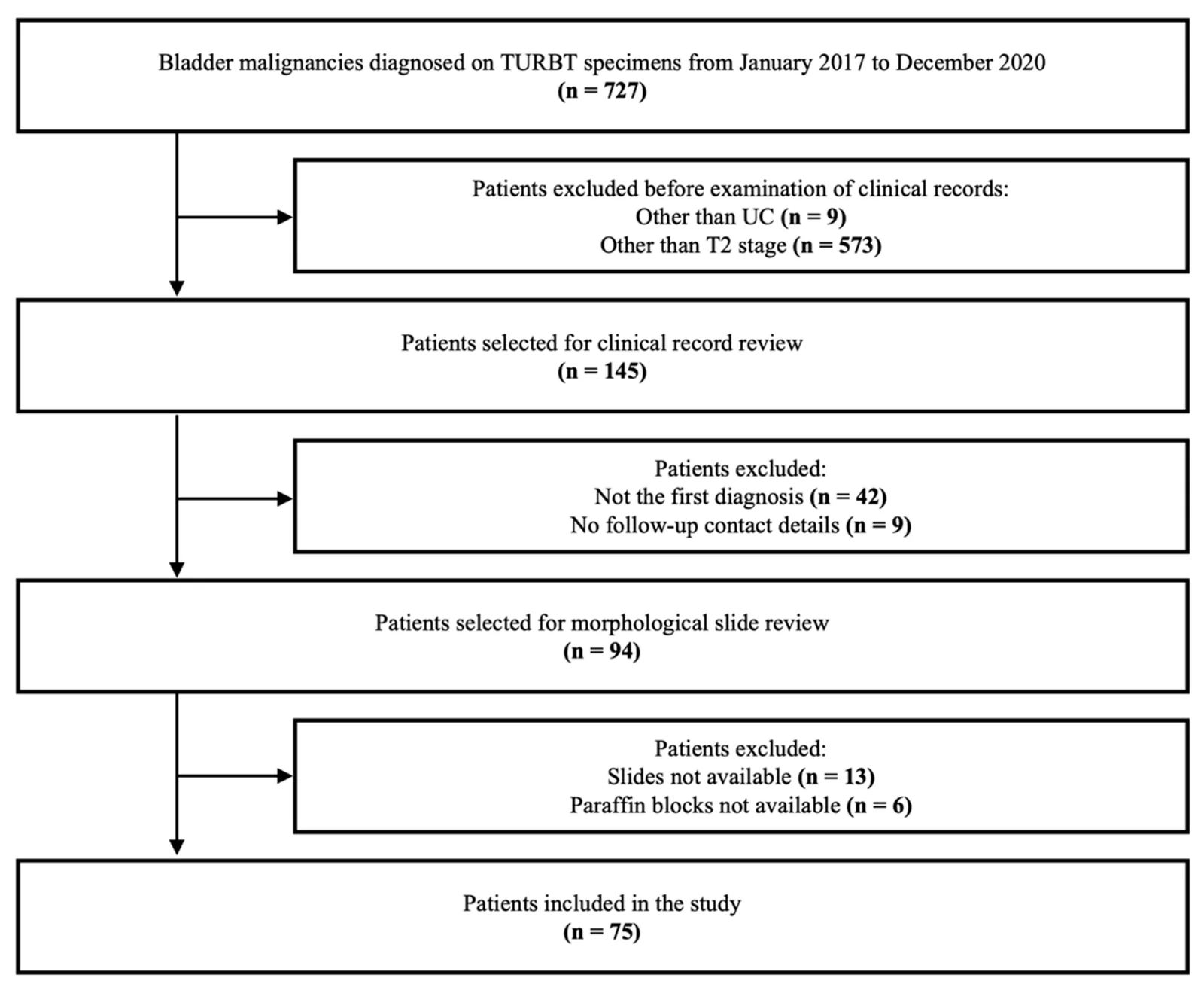
Flow diagram of patient selection based on inclusion and exclusion criteria.

Patients with recurrent disease, non-urothelial histology, absence of muscularis propria invasion, insufficient tumour tissue for IHC analysis, or lack of follow-up or contact information were excluded from the study cohort. The flow diagram in Figure 3 illustrates the inclusion and exclusion criteria and the resulting study cohort.

### 4.4. IHC Analysis

IHC was performed on 4 μm thick FFPE sections using a Bond-Max fully automated immunostainer (Leica Biosystems, Deer Park, IL, USA). The following antibodies were used: GATA3 (clone L50-823, mouse monoclonal, RTU, Agilent, Santa Clara, CA, USA), CK5/6 (clone D5/16 B4, mouse monoclonal, RTU, Agilent, Santa Clara, CA, USA), p16 (clone 6H12, mouse monoclonal, RTU, Leica Biosystems, Deer Park, IL, USA), FGFR3 (ab10651, rabbit polyclonal, dilution 1:1000, Abcam, Cambridge, UK), and EpCAM (clone Ber-EP4, mouse monoclonal, RTU, Agilent, Santa Clara, CA, USA). All procedures were carried out according to the manufacturers’ instructions. Positive IHC control tissues included urothelium for GATA3, skin for CK5/6, high-grade squamous intraepithelial cervical lesion (HSIL) for p16, seminiferous epithelium for FGFR3, and renal collecting ducts for EpCAM. Negative controls were obtained by omission of the primary antibody.

Three pathologists (ACT, AL, AB), blinded to the patients’ clinical outcomes, independently evaluated the IHC slides. Scoring was restricted to the muscle-invasive component of the tumour. For each case, the proportion of positive tumour cells (0–100%, in 10% increments) and staining intensity (0–3; 0 = no staining, 1 = weak, 2 = moderate, 3 = strong) were assessed. IHC positivity was recorded as nuclear (GATA3), cytoplasmic (CK5/6), both nuclear and cytoplasmic (p16), cytoplasmic and membranous (FGFR3), or exclusively membranous (EpCAM). For GATA3 and CK5/6, cases were considered positive when ≥20% of tumour cells showed at least moderate (2+) staining intensity [41]. For p16, positivity was defined as the presence of both nuclear and cytoplasmic staining in ≥70% of tumour cells [18,42]. For FGFR3 and EpCAM, any detectable expression in tumour cells was considered positive [43,44,45,46].

### 4.5. Molecular Classification

**Figure 4:**
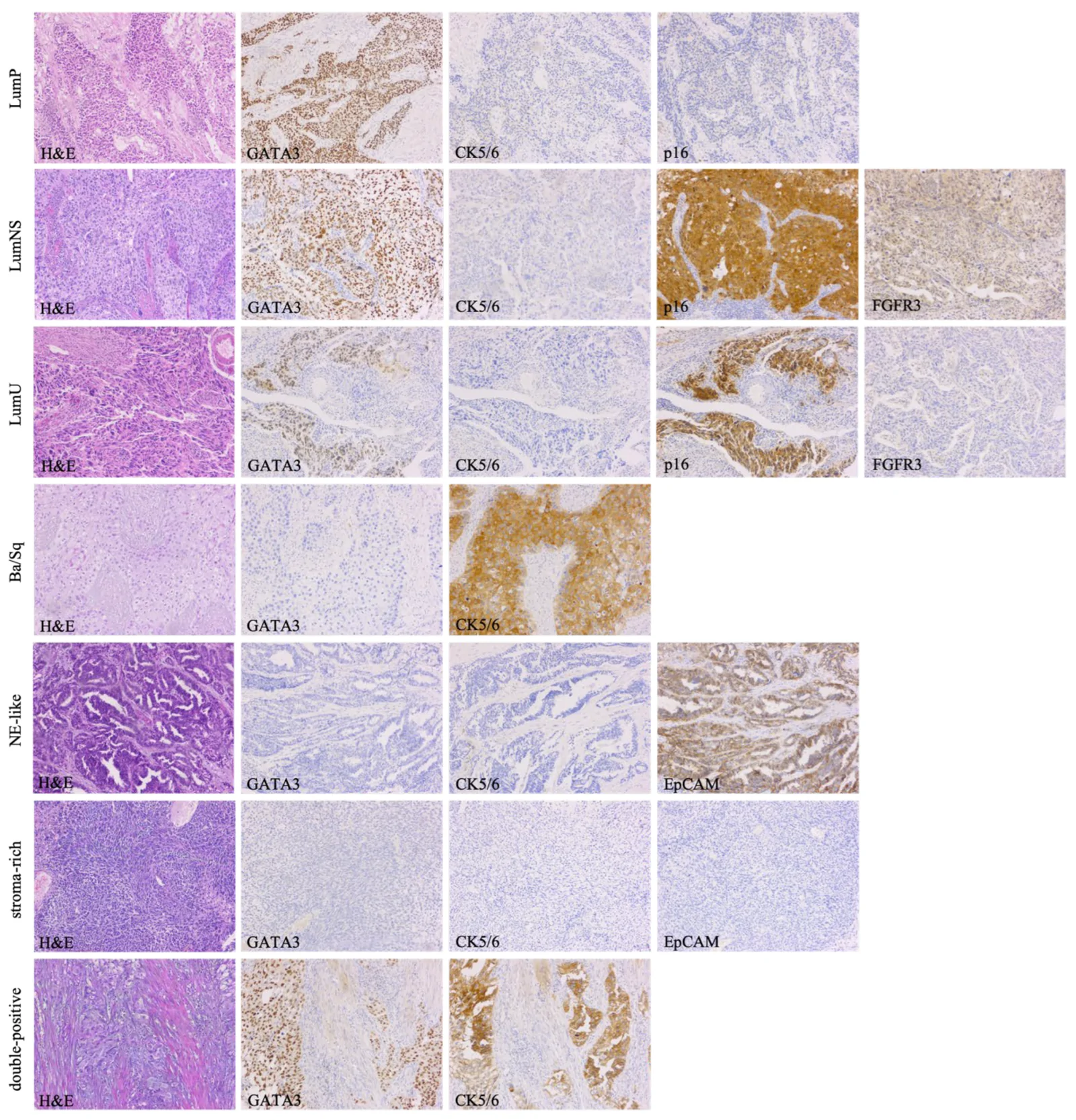
IHC-based molecular classification of MIBUC.

According to the predefined IHC algorithm, tumours positive for GATA3 and negative for CK5/6 were classified as luminal, whereas those negative for GATA3 and positive for CK5/6 were classified as Ba/Sq. Cases exhibiting co-expression of GATA3 and CK5/6 were categorized as double-positive, while those lacking expression of both markers were defined as double-negative. Within the luminal subgroup, p16 expression was used to distinguish LumP (p16-negative) from LumNS and LumU (both p16-positive), while FGFR3 expression further differentiated LumNS (FGFR3-positive) from LumU (FGFR3-negative) [42,47,48]. Within the double-negative group, EpCAM positivity identified the NE-like subtype, whereas EpCAM-negative cases were classified as stroma-rich [19]. Representative IHC staining patterns corresponding to each molecular subtype are shown in Figure 4.

### 4.6. Follow-Up Data

Follow-up data for OS were collected. OS was defined as the time from diagnosis to death from any cause or to the last follow-up. Patients alive at 5 years after diagnosis were censored at the date of their last follow-up.

### 4.7. Statistical Analysis

Statistical analyses were performed using IBM SPSS Statistics version 26.0 (IBM Corp., Armonk, NY, USA). Categorical variables were expressed as frequencies and percentages and compared using the Pearson chi-square or Fisher’s exact test, while continuous variables were reported as medians with interquartile ranges (IQRs). OS was estimated using the Kaplan–Meier method and compared between groups with the log-rank test. A two-sided *p* value < 0.05 was considered statistically significant.

## 5. Conclusions

In conclusion, a five-antibody IHC panel represents a feasible approach for molecular classification of MIBUC at diagnosis and may complement conventional histopathological assessment by capturing additional biological heterogeneity. Although survival differences among molecular subtypes did not reach statistical significance in our cohort, the observed patterns warrant further investigation of the potential prognostic relevance of this classification. Larger, independent, multicentre studies are needed to determine whether IHC-based molecular classification may have clinical utility for prognostic assessment or treatment stratification.

## Figures and Tables

**Table 1:** Patient and tumour characteristics according to molecular subtypes.

Molecular Subtype (n, %)	Median Age at Diagnosis, Years (IQR)	Gender, Number (%)		Histology, Number (%)		Vital Status at Study End-Point, Number (%)		Median Time to Death, Months (IQR)
		M	F	Conventional UC	Variant Histology	Alive	Deceased	
LumP (15, 20%)	71.0 (66.5–80.5)	14 (93.3%)	1 (6.6%)	12 (80.0%)	3 (20.0%)	1 (6.6%)	14 (93.3%)	17.0 (12.0–25.0)
LumNS (13, 17.3%)	72.0 (67.0–78.0)	13 (100%)	0 (0%)	12 (92.3%)	1 (7.7%)	2 (15.4%)	11 (84.6%)	9.0 (8.0–23.0)
LumU (16, 21.3%)	65.5 (62.8–72.3)	15 (93.8%)	1 (6.2%)	13 (81.3%)	3 (18.7%)	3 (18.8%)	13 (81.2%)	15.0 (12.0–33.0)
Ba/Sq (7, 9.3%)	73.0 (55.5–82.0)	6 (85.7%)	1 (14.3%)	3 (42.9%	4 (57.1%)	2 (28.6%)	5 (71.4%)	10.0 (7.0–12.0)
NE-like (5, 6.7%)	72.0 (71.0–78.0)	5 (100%)	0 (0%)	3 (60.0%)	2 (40.0%)	1 (20.0%)	4 (80.0%)	5.0 (4.5–5.5)
stroma-rich (5, 6.7%)	74.0 (73.0–79.0)	5 (100%)	0 (0%)	4 (80.0%)	1 (20.0%)	0 (0%)	5 (100%)	26.0 (2.0–26.0)
double-positive (14, 18.7%)	69.0 (66.5–78.8)	11 (78.6%)	3 (21.4%)	8 (57.1%)	6 (42.9%)	4 (28.6%)	10 (71.4%)	11.0 (4.8–26.8)

**Table 2:** Median survival and estimated survival rates with 95% CIs according to age, sex and tumour morphology.

Clinicopathological Parameter	Median OS, Months (95% CI)	1-Year Survival Rate, % (95% CI)	2-Year Survival Rate, % (95% CI)	5-Year Survival Rate, % (95% CI)	Log-Rank *p*-Value
≤65 years	25 (2.6–47.4)	76.2 (66.9–85.5)	47.6 (36.7–58.5)	33.3 (23.0–43.6)	0.024
W>65 years	12 (7.9–16.1)	50.0 (43.2–56.8)	35.2 (28.7–41.7)	11.1 (6.8–15.4)	
Men	16 (6.7–25.3)	56.5 (50.5–62.5)	42.0 (36.1–47.9)	17.4 (12.8–22.0)	0.626
Women	16 (0.0–52.0)	66.7 (47.5–85.9)	50.0 (29.6–70.4)	16.7 (1.5–31.9)	
Conventional UC	16 (6.7–25.3)	55.0 (43.9–66.1)	40.0 (29.0–51.0)	20.0 (11.1–28.9)	0.884
UC with variant histology	16 (0.7–31.3)	58.2 (51.5–64.9)	38.2 (31.6–44.8)	16.4 (11.4–21.4)	

OS = overall survival; CI = confidence interval.

**Table 3:** Median survival and estimated survival rates with 95% CIs for the seven molecular subtypes.

Molecular Subtype	Median OS, Months (95% CI)	1-Year Survival Rate, % (95% CI)	2-Year Survival Rate, % (95% CI)	5-Year Survival Rate, % (95% CI)	Log-Rank *p*-Value
LumP	18 (7.9–28.1)	66.7 (54.5–78.9)	26.7 (15.3–38.1)	6.7 (0.3–13.1)	0.714
LumNS	12 (0.6–23.5)	46.2 (32.4–60.4)	30.8 (18.0–43.6)	15.4 (5.4–25.4)	
LumU	16 (0.3–31.7)	75.0 (64.2–85.8)	43.8 (31.4–56.2)	18.8 (9.0–28.6)	
Ba/Sq	12 (6.9–17.1)	42.9 (24.2–61.6)	28.6 (11.5–45.7)	N/A	
NE-like	5 (2.9–7.1)	20.0 (2.1–37.9)	N/A	N/A	
stroma-rich	26 (5.0–47.0)	60.0 (38.1–81.9)	20.0 (2.1–37.9)	0.0 (0.0–0.0)	
double-positive	26 (0.0–55.3)	57.1 (43.9–70.3)	50.0 (36.6–63.4)	28.6 (16.5–40.7)	

OS = overall survival; CI = confidence interval; N/A = not applicable.

## Data Availability

Dataset available on request from the authors.
